# Background-Free Near-Infrared Biphoton Emission from Single GaAs Nanowires

**DOI:** 10.1021/acs.nanolett.3c00026

**Published:** 2023-04-14

**Authors:** Grégoire Saerens, Thomas Dursap, Ian Hesner, Ngoc M. H. Duong, Alexander S. Solntsev, Andrea Morandi, Andreas Maeder, Artemios Karvounis, Philippe Regreny, Robert J. Chapman, Alexandre Danescu, Nicolas Chauvin, José Penuelas, Rachel Grange

**Affiliations:** † ETH Zurich, Department of Physics, Institute for Quantum Electronics, Optical Nanomaterial Group, 8093 Zurich, Switzerland; ‡ Univ. Lyon, CNRS, ECL, INSA Lyon, UCBL, CPE Lyon, INL, UMR 5270, 69130 Ecully, France; § University of Technology Sydney, School of Mathematical and Physical Sciences, Ultimo, New South Wales 2007, Australia

**Keywords:** spontaneous parametric down-conversion, second-harmonic generation, III−V semiconductors, GaAs nanowires, room temperature

## Abstract

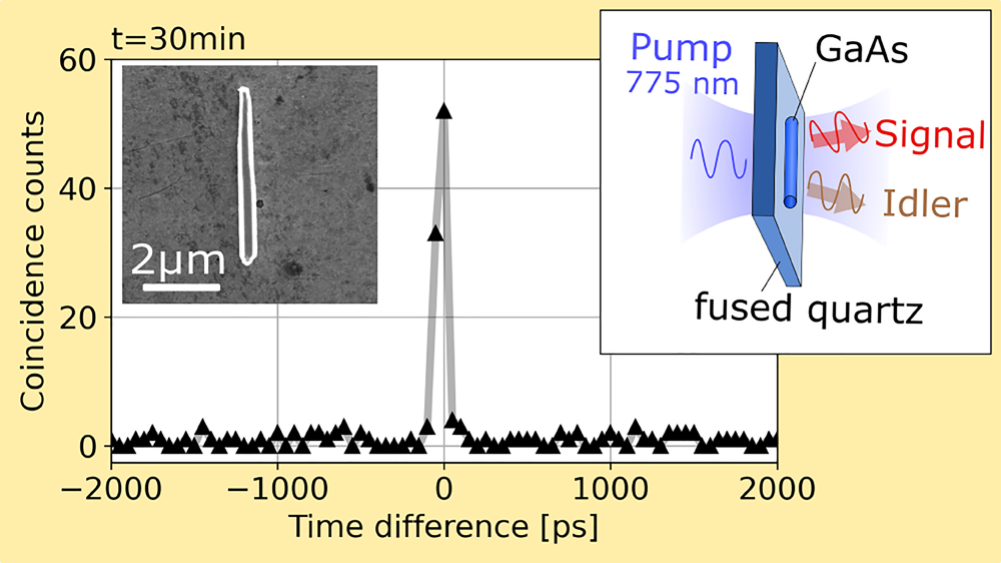

The generation of photon pairs from nanoscale structures with high rates is still a challenge for the integration of quantum devices, as it suffers from parasitic signals from the substrate. In this work, we report type-0 spontaneous parametric down-conversion at 1550 nm from individual bottom-up grown zinc-blende GaAs nanowires with lengths of up to 5 μm and diameters of up to 450 nm. The nanowires were deposited on a transparent ITO substrate, and we measured a background-free coincidence rate of 0.05 Hz in a Hanbury–Brown–Twiss setup. Taking into account transmission losses, the pump fluence, and the nanowire volume, we achieved a biphoton generation of 60 GHz/Wm, which is at least 3 times higher than that of previously reported single nonlinear micro- and nanostructures. We also studied the correlations between the second-harmonic generation and the spontaneous parametric down-conversion intensities with respect to the pump polarization and in different individual nanowires.

Miniaturization of biphoton light sources is an important requirement to achieve scalable and stable integrated quantum devices.^1^ Photon pairs, or similarly heralded single photons, generated via spontaneous parametric down-conversion (SPDC) profit from high indistinguishability, room-temperature operation, simple signal filtering, and coherent emission as well as entanglement in several degrees of freedom.^2−4^ Applications have been demonstrated in different quantum technologies, including communication,^5−10^ imaging,^11−13^ computing,^14−16^ and metrology.^17−19^ Despite its intrinsically limited efficiency to avoid multiphoton events, compared, for example, to quantum dot- or atom-based photon sources,^20,21^ the brightness of SPDC-based photon sources can be improved with multiplexing without an increase in unwanted double-photon pairs.^1,22,23^

Today’s leading nonlinear light sources are bulk birefringent or periodically poled crystals^24−26^ and periodically poled waveguides,^27−30^ with millimeter length scales, for which phase matching is required. Miniaturization to the micrometer or submicrometer scale, to sizes smaller than the coherence length, relaxes the phase-matching condition and reduces the footprint of the source.^31^ SPDC with no phase-matching requirements has recently been demonstrated in thin films^32−34^ and metasurfaces,^35−38^ for example by taking advantage of resonances such as bound states in the continuum or Mie resonances. A single nanoantenna does not need phase matching either,^39^ and arrays of nanoantennas can be collectively excited for an increased generation rate. To our knowledge, SPDC generation from a single structure has only been demonstrated at 1550 nm in two different types of geometries, top-down AlGaAs nanoresonators^40^ and freestanding LiNbO_3_ microcubes.^41^ However, this has not been demonstrated in GaAs nanostructures despite the strong χ^(2)^ tensor (*d*_36_ = 370 pm/V^42^), high refractive index (3.4 for λ around 1550 nm^43^), and various fabrication methods.

Here we report type-0 SPDC in individual self-assisted grown zinc-blende (ZB) GaAs nanowires (NWs) with lengths of 5.2 ± 0.4 μm and diameters of 430 ± 30 nm. Due to the high tensor components of the GaAs crystal structure compared to other typical nonlinear materials (β-barium borate, potassium dihydrogen phosphate, or lithium niobate), and the possibility to place the NW on a transparent substrate, we obtained at room temperature and for 15 mW incident power a high photon-pair rate of 0.05 Hz with a corresponding coincidences-to-accidentals ratio (CAR) of 60 in the coincidence histogram. The efficiency given as a transmission-corrected photon pair rate normalized to the excitation fluence and nanostructure volume was 60 GHz/Wm. This is 40 times higher than that of AlGaAs nanoresonator^40^ and 3 times higher than that of LiNbO_3_ microcube systems.^41^

Self-assisted GaAs NWs with a ZB crystal structure and a slight Be p-doping were grown by molecular beam epitaxy on a silicon (111) substrate. It should be noted that the NWs also possess a short defective region at the bottom and at the top, including a wurtzite (WZ) segment at the top.^44^ After growth, the NWs were mechanically transferred to a transparent thin ITO layer (10 nm) on an SiO_2_ substrate. The detailed fabrication steps are given in section 1 of the Supporting Information. In section 2 of the Supporting Information, we characterized the NWs optically. We show on the one hand that the NWs are nonresonant around 775 and 1550 nm by collecting the scattering spectrum using a dark field microscope. On the other hand, we confirm optically the crystal uniformity in the middle of the NWs by imaging the dependence of the second-harmonic-generation (SHG) intensity with the linear polarization of the incoming pump laser.^45^ We recorded SPDC with a Hanbury–Brown–Twiss (HBT) setup as shown in Figure 1. First, a linearly polarized continuous-wave laser with a wavelength of 775 nm and 15 mW power was focused with a lens (*f* = 8 mm) on a single NW. The generated photon pairs were then collected with a 50× NIR objective (*f* = 4 mm) and separated from the pump with two low-pass filters. After that, the light was coupled to a 1550 nm fiber, split in two channels, and finally detected with superconducting nanowire single-photon detectors (SNSPDs). A time-to-digital converter (TDC) was used to measure coincidence counts between the two channels. A 1550 nm laser was used for the SHG process and to maximize the fiber transmission and detection efficiency. In section 3 of the Supporting Information, we describe the setup in detail and give the alignment procedure to measure photon pairs. We discuss in section 4 of the Supporting Information the impact of the pump wavelength, which is smaller than the band gap of GaAs.

**Figure 1:**
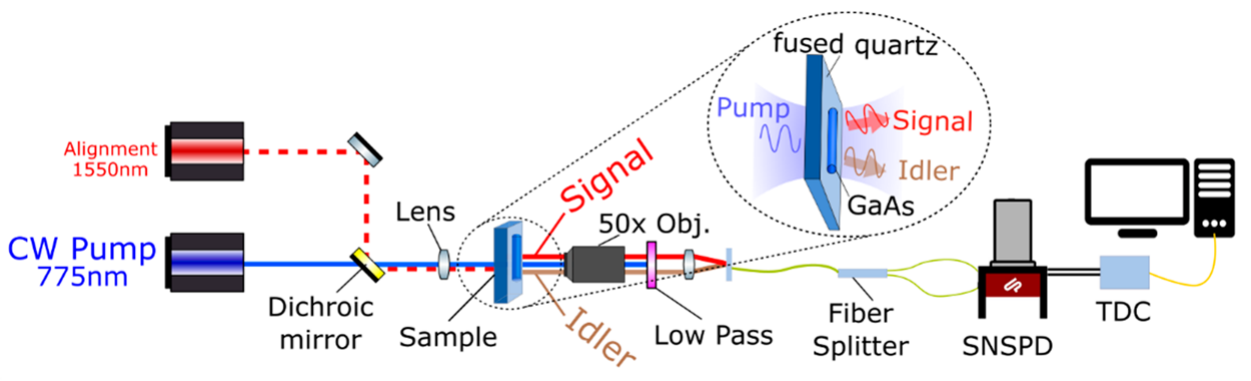
Hanbury–Brown–Twiss (HBT) setup using a continuous-wave pump laser with a wavelength of 775 nm. The photon pairs are collected with a 50× objective and filtered with two low-pass filters. Photons are detected with superconducting nanowire single-photon detectors (SNSPDs), and the coincidence counts of two channels are measured using a time-to-digital converter (TDC). The transmission in the fiber and the detection efficiency are both maximized using a 1550 nm laser.

We excite a GaAs NW with a length of 5 μm and a diameter of 450 nm using a linearly polarized laser along its crystal axis, with a spot size of around 6 μm radius (Figure 2a). The measured coincidence counts over 30 min show a photon pair rate of 0.05 Hz (90 counts over 30 min, see Figure 2b) with a CAR equal to 60, which should be greater than 2 for photon pairs. The accidental counts are much lower than the signal counts because the NW is lying on a transparent substrate. The SPDC process is also confirmed by the linear increase in photon pairs with respect to the incoming power, as shown in Figure 2c, indicating that only one photon is involved as a pump in the nonlinear process. Phase matching in such nanostructures is not necessary, as the coherence length of GaAs is around 2.7 μm, which is much more than the thickness of the NW (see calculation in section 4 of the Supporting Information).

**Figure 2:**
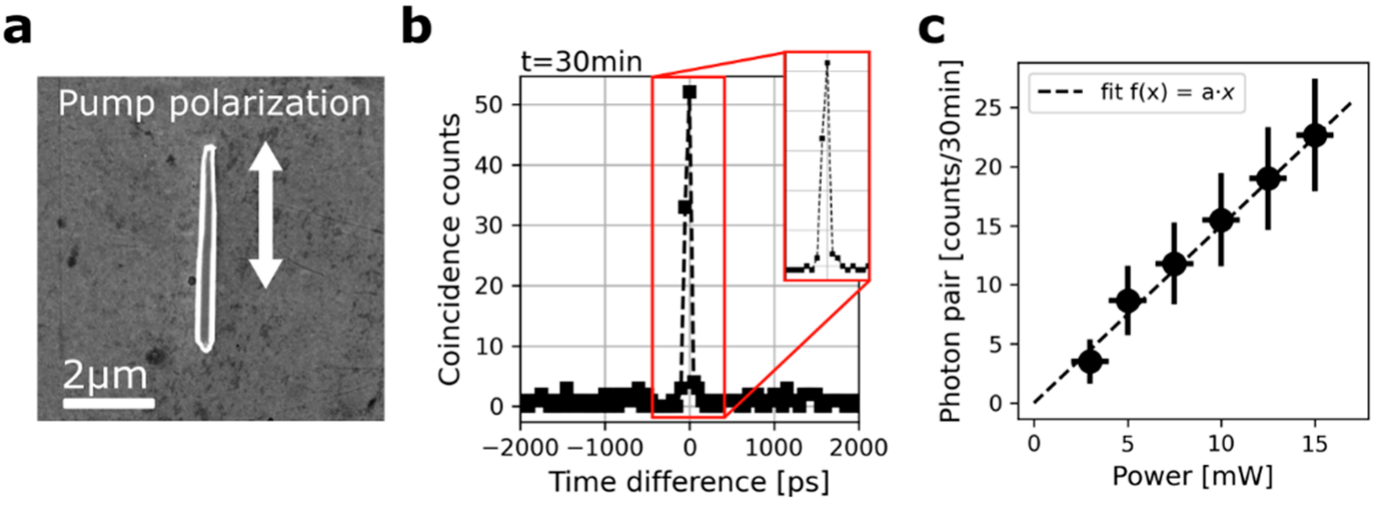
(a) Scanning electron microscopy image (SEM) of the GaAs nanowire (NW) with a length of 5 μm and a diameter of 450 nm. The linear polarization of the pump, which is along the long axis of the NW, is indicated by the white arrow. (b) Coincidence counts measured over 30 min with a maximum count of 52 at zero-time difference and a coincidences-to-accidentals ratio (CAR) well above 2. The detected biphoton arrivals is calculated as the sum of the coincidence counts around zero-time difference, which is 90 here. (c) Linear dependence of photon-pair counts with incoming pump power.

From the quantum-classical correspondence relation between SPDC and SHG as depicted in Figure 3a,^46^ we expected a similar polarization dependence of the second-order nonlinear signals for nonresonant structures,^41^ and we could indeed observe it. The SPDC (SHG) intensity measured for different polarizations of the 775 nm (1550 nm) pump laser is indicated by red circles (blue diamonds) in Figure 3b. A maximum SPDC (SHG) signal intensity was obtained for a linear polarization along the NW. In addition, we characterized the polarization of the photon pairs by placing a linear polarizer before the fiber coupling. The linear polarization of the pump was fixed along the NW, for which the photon-pair rate without polarizer was the highest, as shown previously in Figure 2b. Figure 3c shows in red the photon pair counts without a polarizer and for different orientations of the polarizer. We measured a maximum photon-pair rate for the polarizer oriented along the NW and no signal for the polarizer oriented perpendicularly to the NW. As a matter of fact, the maximum rate was only (90%)^2^ of that of the biphoton rate without polarizer, which corresponds to the transmission losses of photon pairs from the polarizer. We compared the impact of the polarizer’s rotation on the photon-pair rate (red points) directly with the transmission squared of a 1550 nm laser (blue curve), for which the linear polarization was the same as the SPDC pump laser. We observed a very good matching; as shown in Figure 3c, the NW emits photon pairs with the same polarization as the excitation light, along the NW’s axis. We thus conclude that the SPDC is type-0 for NWs pumped along the long axis. Similar polarization-dependent Raman scattering, SHG, or single-photon emission have already been observed from single NWs.^45,47−50^ Considering the crystal structure orientation of our GaAs NWs, in our case, the dependence of SHG and SPDC on the pump polarization matched the expectation from our calculation of the χ^(2,*i*)^ tensor in the rotation frame *i* = 1, 2, 3. In section 5 of the Supporting Information we show similar polarization-dependent SHG emission profiles for 14 different NWs. The χ_*x,x,x*_^(2)^ value leads indeed to a strong SHG emission intensity for *x*-polarization, which is along the NW’s axis. The slight mismatch between the polarization measurement shown in Figure 3b,c can be understood from the alignment procedure, which involves multiple waveplates (see section 3 in the Supporting Information).

**Figure 3:**
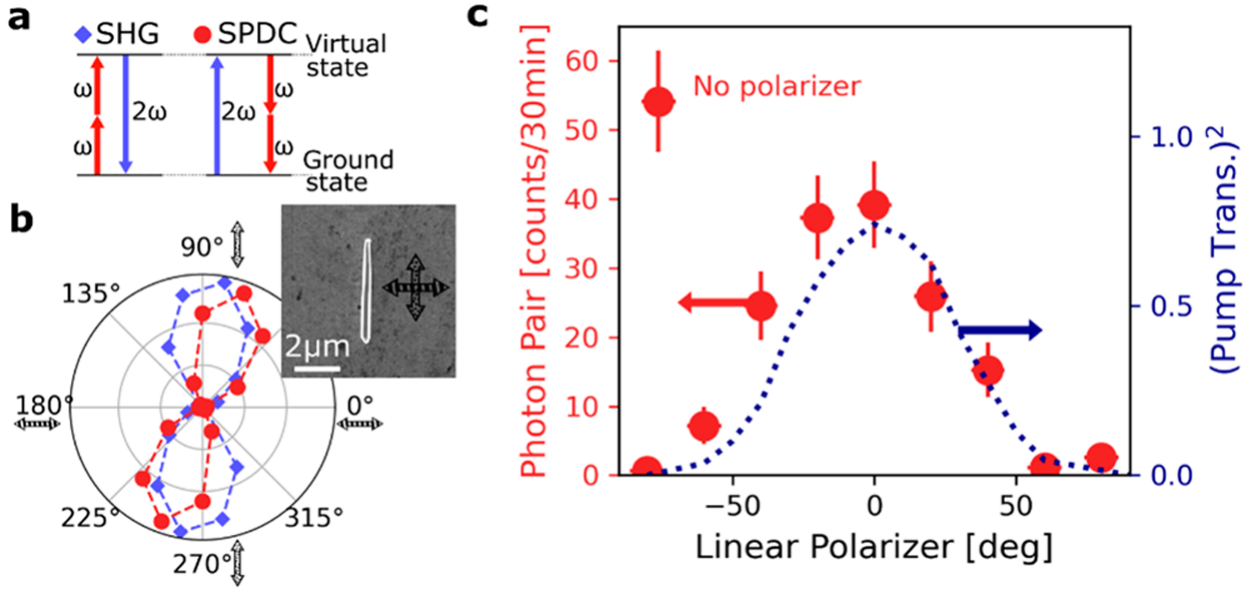
(a) Schematic of energy transitions involved in the SHG and SPDC process. (b) Normalized SHG (blue diamonds) and SPDC (red circles) signal for different linear polarization orientations of the pump. The inset shows the same SEM image of the NW as in Figure 2a. The lighter (darker) arrow corresponds to pump photons linearly polarized at 90°/270° (0°/180°) with respect to the horizon (laboratory reference frame). (c) Similar measurement at a fixed pump polarization (90°/270°) with a linear polarizer set before the fiber in-coupling. We give with red dots the photon pairs measured for different orientations of the polarizer and with the blue dotted line the expected squared transmission from photons polarized along the NW’s axis, as the 775 nm pump laser.

Finally, we measured similarly the second-order nonlinear intensities (SHG and SPDC) from 14 single GaAs NWs. We observed a linear correlation between SHG intensity and biphoton rates, as shown in Figure 4. NWs generating low SHG intensities also emit low photon-pair rates. We noticed that the nonlinear emission intensity did not depend on the NW’s size: see for example NW no. 10 in Figure 4 with a higher volume compared to the average but with lower nonlinear intensities. We confirmed this dependence by analyzing more in detail the impact of the NW’s size on the biphoton rate (see section 6 of the Supporting Information). We also evaluated for each NW if their linear optical properties were correlated with the nonlinear emission. We found for each NW that the linear optical properties are not strongly correlated with the nonlinear emission. When taking into account the losses in the setup (objective transmission, fiber in-coupling, fiber splitter, and detector), we find a photon-pair rate of up to 20 Hz. This correction does not take into account photon pairs emitted backward or forward but only collected by a NA = 0.65 objective. In order to compare with other miniaturized sources, we normalized this transmission-corrected rate as in Marino et al.^40^ with the volume of the NW and the pump fluence. The resulting efficiency of the SPDC process can reach 60 GHz/Wm for GaAs NWs (see purple scale bar in Figure 4a). The detailed calculations and the numerical values of the efficiencies are given in section 6 of the Supporting Information. The overall crystal structure uniformity is shown in Figures S3 and S4 of the Supporting Information, but nanoscopic crystal structure defects or doping concentration variations may be present and could still have some impact on the biphoton emission efficiency. In comparison, the SPDC efficiency from GaAs NWs is 40 times higher than that of similar AlGaAs nanoantennas^40^ and 3 times higher than that of LiNbO_3_ microcubes.^41^ We consider this was possible thanks to the NW’s growth and transfer method and the geometry of the system, the one-dimensionality of the NW, leading to an accessible and strong *d*_36_ component of the bulk GaAs ZB χ^(2)^ tensor.

**Figure 4:**
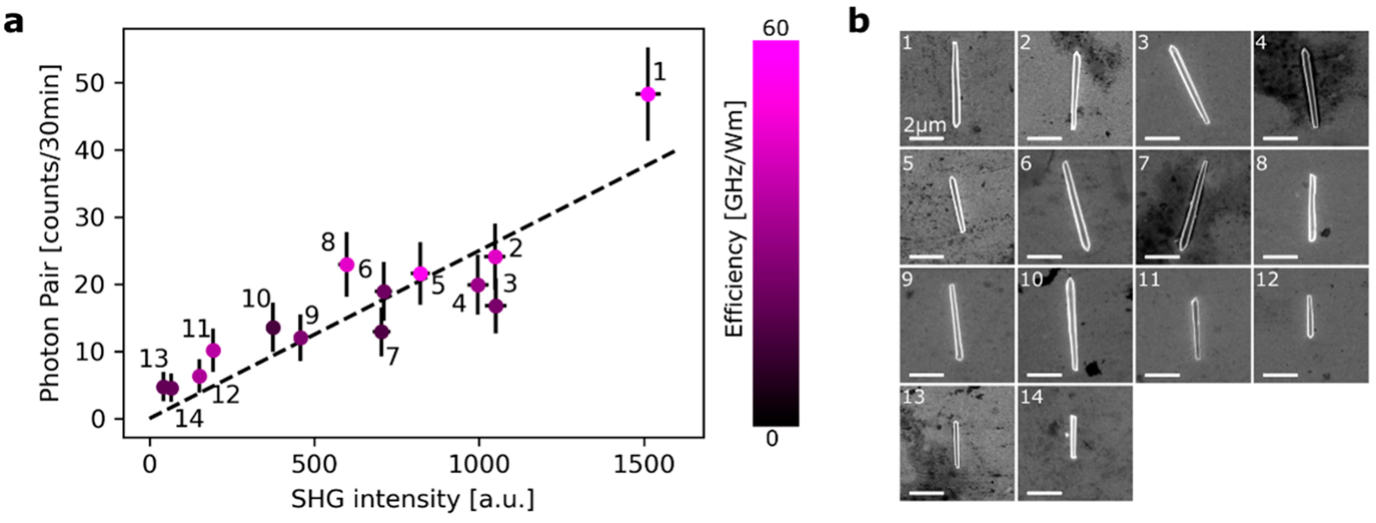
(a) Correlation between SHG (*x*-axis) and SPDC (*y*-axis) intensity for 14 different NWs. The measured biphoton rates normalized to the transmission losses, volume, and (fixed) pump fluence are given as efficiencies with the pink scale bar. The maximum calculated efficiency was 60 GHz/Wm. (b) SEM images of the 14 single NWs.

## Conclusion

We have investigated type-0 photon pairs generated at 1550 nm wavelength from SPDC in single GaAs NWs. We have tested the quantum-classical correspondence by comparing on the one hand the pump polarization impact on the SHG intensity with the SPDC rate and on the other hand the nonlinear generation from 14 similar NWs. We observed a linear correlation between SPDC and SHG intensities. For individual self-assisted grown ZB GaAs NWs with 5 μm length and 450 nm diameter we obtained a maximum photon pair rate of 20 Hz. We have demonstrated GaAs NWs as photon pair sources profiting from stability, time coherence, and room-temperature operation. These structures could be directly grown on a photonic chip or transferred and freely positioned on it using an atomic force microscopy tip. They would act as quantum light emitters that do not need quantum confinement engineering. Further study into the geometry and crystal orientation of III–V nanostructures would benefit research not only in nonlinear processes such as SHG and SPDC but also in single-photon emitters such as defects and quantum dots.

## Supporting Information Available

Details of fabrication, nanowire optical characterization, setup, and alignment to measure SHG and SPDC, the GaAs coherence length, the GaAs bulk χ^(2)^ tensor specific to this experiment, and a summary of the NW efficiency (PDF)

## Abbreviations

NW: nanowire
SPDC: spontaneous parametric down-conversion
SHG: second-harmonic generation
ZB: zinc-blende
CAR: coincidences-to-accidentals ratio
HBT: Hanbury–Brown–Twiss
SNSPD: superconducting nanowire single-photon detector
TDC: time-to-digital converter
