# Supplementary material for: Background-Free Near-Infrared Biphoton Emission from Single GaAs Nanowires
Source: Nano Lett. 2023 Apr 14;23(8):3245–50. doi: 10.1021/acs.nanolett.3c00026 (PMC10141417; doi:10.1021/acs.nanolett.3c00026)
Supplement: Supplementary file 1 — nl3c00026_si_001.pdf [file nl3c00026_si_001.pdf]

Supplementary Material: Background-Free Near-Infrared Biphoton Emission from Single GaAs Nanowires

Grégoire Saerens[a], Thomas Dursap[b], Ian Hesner[a], Ngoc M. H. Duong[a], Alexander S. Solntsev[c], Andrea Morandi[a], Andreas Maeder[a], Artemios Karvounis[a], Philippe Regreny[b], Robert J. Chapman[a], Alexandre Danescu[b], Nicolas Chauvin[b], José Penuelas[b] and Rachel Grange[a]*

[a] ETH Zurich, Department of Physics, Institute for Quantum Electronics, Optical Nanomaterial Group, 8093 Zurich, Switzerland

[b] Univ. Lyon, CNRS, ECL, INSA Lyon, UCBL, CPE Lyon, INL, UMR 5270, 69130 Ecully, France

[c] University of Technology Sydney, School of Mathematical and Physical Sciences, Ultimo NSW 2007, Australia

Corresponding Author

* gsaerens@phys.ethz.ch

Contents

1. Fabrication 2
2. Nanowire characterization 3
3. Alignment and SPDC/SHG measurement 6
4. Coherence length and absorption in GaAs 9
5. $X^{(2)}$ tensor rotation 9
6. Summary SPDC efficiency calculation 13

1. Fabrication

The self-assisted GaAs Nanowires (NWs) were grown on epi-ready Si(111) substrates using a solid-source molecular beam epitaxy (MBE) reactor. The substrates were systematically cleaned during 5 minutes in both acetone and ethanol, and degassed at 200°C in ultra-high vacuum before introduction inside the MBE reactor. The native SiO_2 oxide of the substrates was preserved to enable the self-assisted growth.[1]

The growth was hence obtained using the vapor-liquid-solid (VLS) mechanism.[2] To form Ga droplets, the substrate was heated to 440°C and 1 monolayer (ML) of Ga was pre-deposited [3,4] at a deposition rate of 0.08 $ML\cdot s^{-1}$, quoted in units of equivalent growth rates of GaAs thin films measured by RHEED oscillations on GaAs substrates.[5] The substrates were then heated up to 580°C, the growth temperature. Finally, the growth of the NWs was initiated with the simultaneous opening of the Ga and As fluxes. The NWs were grown during 30 min with a Ga and As_4 flux of 0.5 $ML\cdot s^{-1}$ and 1.2 $ML\cdot s^{-1}$, respectively, corresponding to a V/III flux ratio of 2.4. The growth was terminated by the closing of the Ga shutter only, to consume the Ga-catalyst droplet. A radial growth was then performed at 450°C during 60 min to increase the diameter of the NWs.

Finally, the NWs were mechanically transferred on a quartz substrate covered with a 10 nm thin ITO layer on top to enable imaging with SEM, similarly as in our previous work.[6,7] The different fabrication steps are shown schematically in Figure S1 with SEM images in the inset for the

vertically grown NWs and the transferred ones. The NWs have an hexagonal cross-section with a preferred (111) growth axis and their lengths and diameters are defined as in the schematic of Figure S1d.

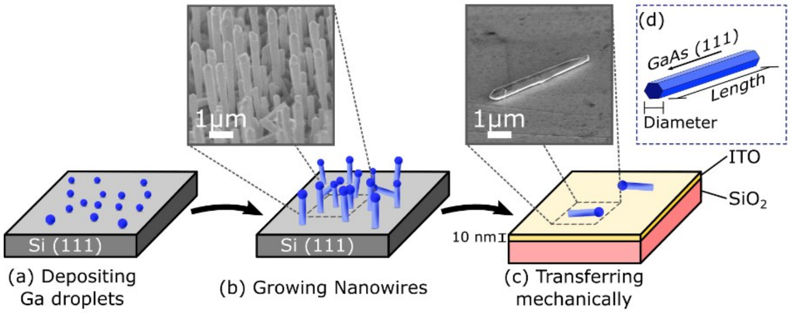

Figure S1. (a)-(b) Self catalyzed process to grow GaAs NWs on (111) Si. Inset: SEM image of the NWs. (c) Mechanical transfer of the NWs with SEM image in the inset. (d) the NWs have an hexagonal cross section and their lengths and diameters, given in the manuscript, are defined as in this schematic.

2. Nanowire characterization

We characterized the linear optical scattering of the 14 different NWs using a dark-field microscope (Zeiss Axio) in transmission, exciting with unpolarized light and without using any polarizer in the setup, similar as in [6–8]. We used a 50x Epiplan Neofluar objective (Zeiss, NA=0.55) to collect the scattered light (fiber core NA = 0.12). The scattering profiles given in Figure S2 do not show resonances around 775 nm nor 1550 nm for any of the 14 nanowires.

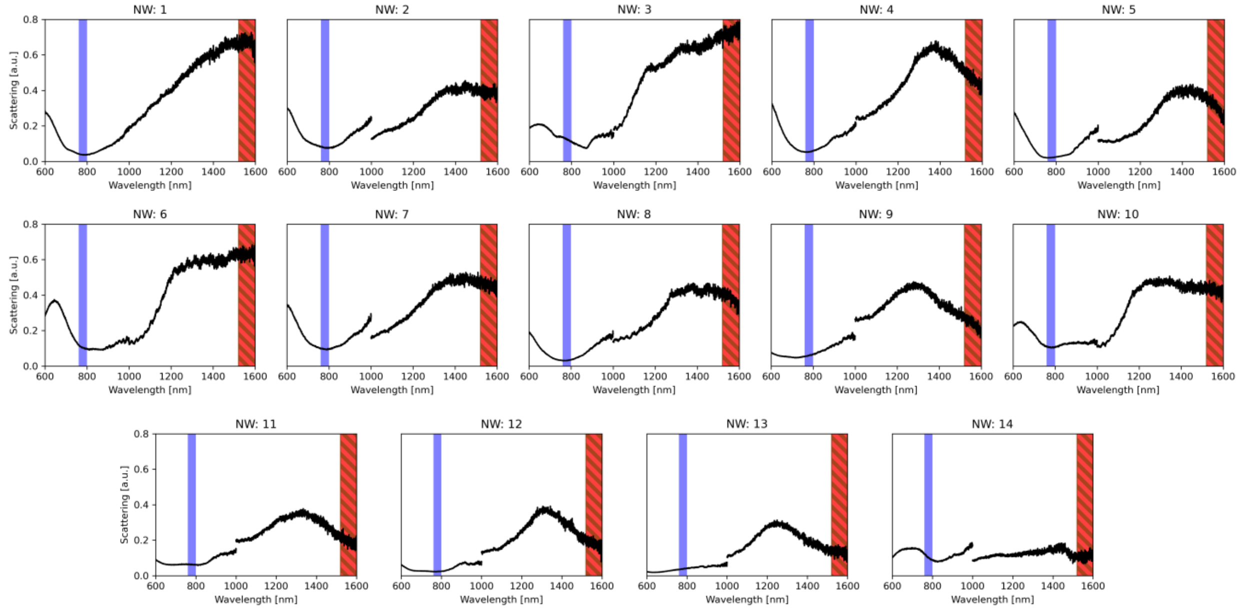

Figure S2. Linear scattering spectra for the 14 single nanowires presented in Figure 4. No resonances are visible in the blue area, indicating the 775 nm wavelength, nor in the red area, indicating the 1550 nm wavelength.

We looked at the second-harmonic generation (SHG) at a fixed wavelength (775 nm) under different pump polarizations for the 14 single NWs. Figure S3a shows a SEM and the SHG intensity images for NW nº1. The total SHG intensity for different pump polarization (polar plot) is plotted for the whole NW in Figure S3b. Figure S3c shows the maximum SHG intensity (in pink) and the polar plots (in blue) for different areas of the NW (black squares).

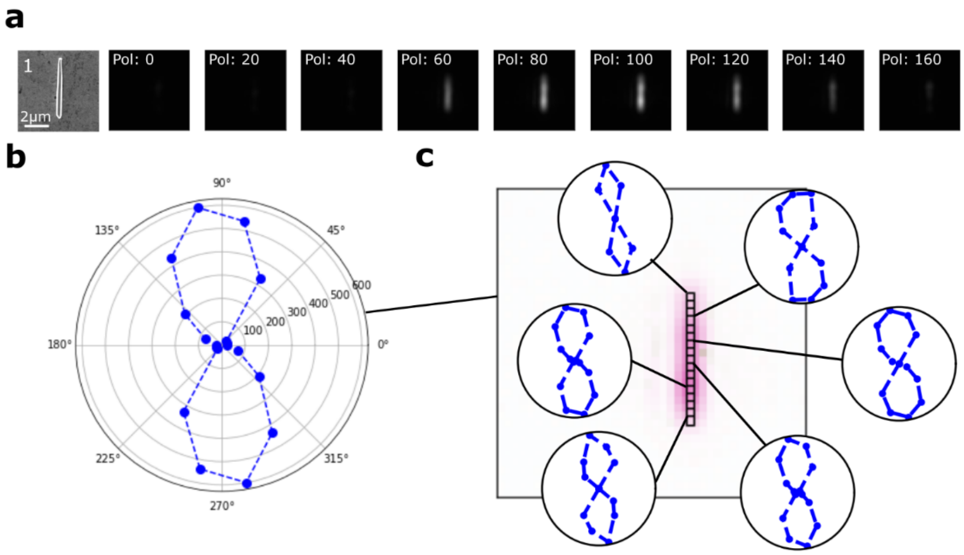

Figure S3. (a) SEM image of the NW first and SHG intensity images for different polarizations. (b) polar plot of the whole NW and (c) polar plot for different regions in the NW. The results always have the same dipolar shape (blue dots) and intensity (purple), indicating a uniform crystal structure.

We confirm the uniform crystal structure in the middle, as the SHG intensity is the strongest at 100° (corresponding to excitation along the NW). Similar results, without showing the extensive polarization dependencies, are shown in Figure S4. All the NWs do show a strong SHG intensity around 100° and a minimum intensity at 0° (similar dipolar shape as Figure S3c).

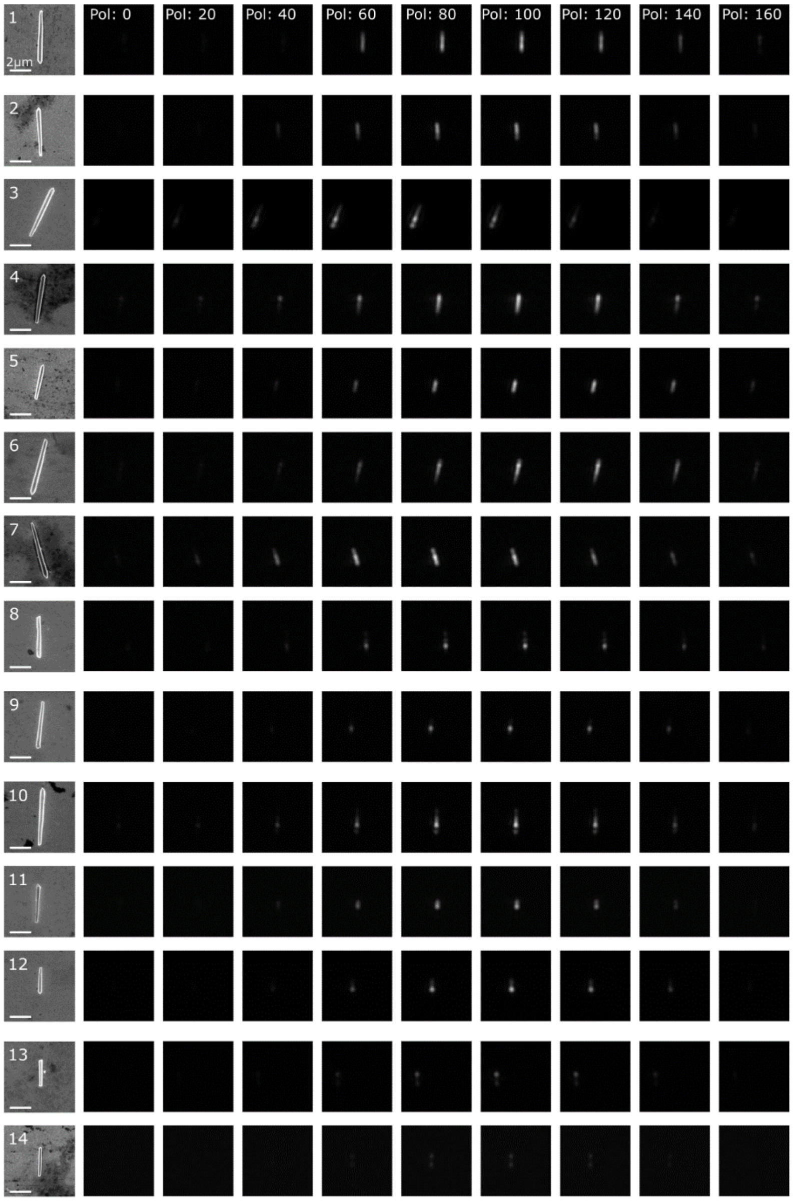

Figure S4. SEM image first and SHG intensity images for different polarizations of each 14 NW (each line one, labeled on the left).

3. Alignment and SPDC/SHG measurement

We performed SPDC and SHG measurements in one setup (see Figure S5), using the same excitation lens (f = 8 mm, NA = 0.5, A240TM from Thorlabs) and collection objective (f = 4 mm, NA = 0.65, M Plan Apo NIR from Mitutoyo). We pumped the NWs for SPDC with a 775 nm continuous laser (Toptica DL pro 780) and for SHG with a pulsed 1550 nm laser (Menhir-1550,

τ=150 fs, R=216 MHz). We filtered the nonlinear signal to cut the pump with two low-pass (LP 1064 + LP 1319 from Semrock) and two high-pass (FESH 900 from Thorlabs) filters for SPDC and SHG respectively. For both type of experiments, the polarization and power, which was always 15 mW unless specified, were set upstream with a combination of λ/2 waveplates and polarized beam splitters and were measured before the excitation lens. Both 775 nm and 1550 nm laser beams were brought together using a dichroic mirror, and aligned collinearly to hit the target. Even though we pumped for SPDC with the 775 nm laser, we used the 1550 nm laser to calibrate the fiber and the superconducting nanowire single photon detector (SNSPD) as they are polarization dependent and designed for 1550nm. The cw laser can be tuned from 760 nm to 800 nm. However, we always pumped at 775 nm, as we believe we would observe similar results at other wavelengths as the ones given here. The NWs are indeed not resonant, and the $\chi^{(2)}$ tensor would only slightly vary (Miller's rule).[9]

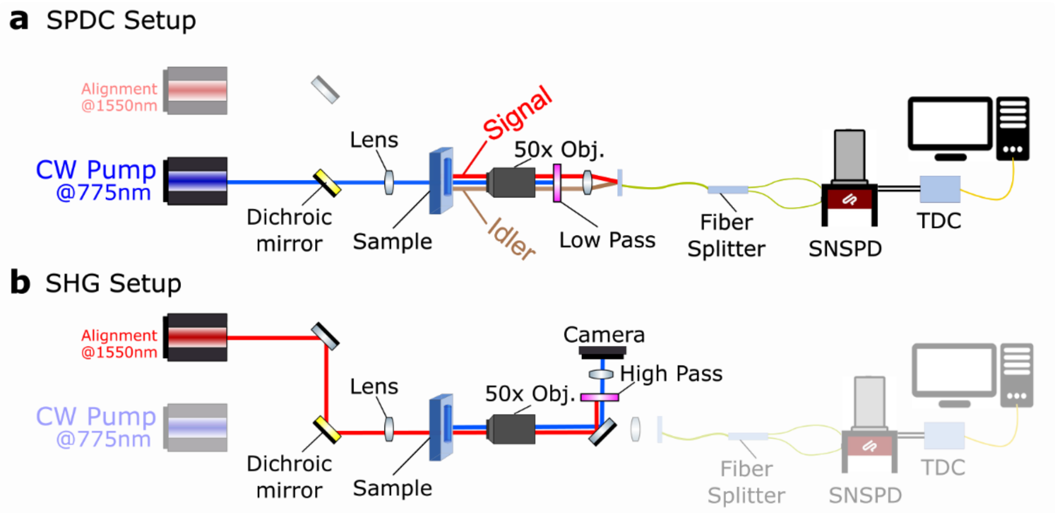

Figure S5. Schematics of the (a) SPDC and (b) SHG setup. The excitation and collection lens are the same for both setups. The pump, filters and detector are different.

The orientation of the λ/2 waveplates to calibrate the polarization and the number of mirrors were carefully selected so that both lasers have a polarization for which 0° corresponds to

horizontal polarization at the sample and 90° to the vertical direction. We used also an additional λ/2 waveplate after the linear polarizer and before the fiber in-coupling for the photon-pair polarization characterization to avoid recalibrating the polarization-sensitive SNSPD detector at each rotation of the polarizer.

The main optical losses in the setup come from the NIR collection objective (60%), the in-coupling to the fiber (75%), the statistical losses from the fiber splitter (50%) and the SNSPD detector (20%). When two photons are propagating in the same fiber channel, the detector will not measure a coincidence, hence the statistical loss from the beam splitter. The coupling into the fiber is done with an adjustable fiber collimator (f = 11 mm, CFC11A-C from Thorlabs) and in this setup the transmission into the fiber can easily reach up to 75% with a collimated beam. However, as the excitation is done at 775 nm and the collected signal is at 1550 nm, the two laser beams cannot simultaneously be collimated. Empirically, the highest SPDC intensity is found when the 1550 nm is closer to the collimation state, giving a 25% coupling transmission into the fiber.

We confirm the SHG process firstly by comparing a spectrum of the pump and the filtered signal and secondly by characterizing the power dependence of the process. Figure S6a shows in red the normalized spectrum of the 1550 nm laser used to pump the NW (ND 8 to attenuate the intensity) and in blue the spectrum of the filtered signal (FESH 900). We observe that the pump laser peak is exactly twice lower in frequency than the SHG signal. The SHG intensity (blue crosses) also scales quadratically with the pump power, which is shown in Figure S6b along with a quadratic fit function (dotted black line). The fluorescence from the NWs was not visible in the spectrometer when pumping at 1550 nm, also the low pass filter would block any fluorescence signal when pumping at 775 nm. We did not either observe fluorescence from GaAs NWs pumped at 775 nm.

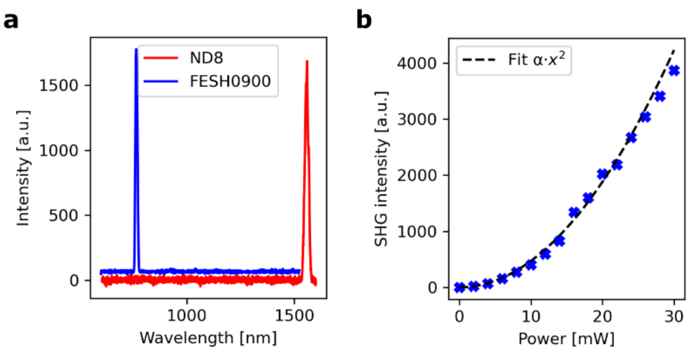

Figure S6: (a) Normalized Spectrum of 1550nm Laser after NW with ND absorber filters (ND8) and of the signal obtained with two high-pass filters (FESH 900). (b) SHG intensity (blue crosses) for different pump powers and a fit with a quadratic function (dotted black line).

4. Coherence length and absorption in GaAs

We calculated the coherence length of GaAs with the following formula $L = \frac{2\pi}{|\Delta k|}$ = 2.7 µm, for which the wave vector difference $\Delta k = \frac{2\pi}{\lambda_p} \cdot n(\lambda_p) - 2 \cdot \frac{2\pi}{\lambda_s} \cdot n(\lambda_s)$ is calculated for λ_p = 775 nm and λ_s = 1550 nm. Also to consider is the absorption of GaAs. At 775 nm wavelength, the absorption coefficient of GaAs is $\alpha = 1.5 \cdot 10^4\ cm^{-1} = 1.5\ \mu m^{-1}$, for which the inverse is still bigger than the diameter D of the NWs, 0.4 µm. Around $T = e^{-\alpha D} \approx 55\%$ of light is transmitted.

5. $X^{(2)}$ tensor rotation

The point group symmetry for the ZB GaAs is $\overline{4}3m$. The $\chi^{(2)}$ tensor to describe second order nonlinear processes is given again here:

$$\chi^{(2)} = \begin{pmatrix} 0 & 0 & 0 & d_{36} & 0 & 0 \\ 0 & 0 & 0 & 0 & d_{36} & 0 \\ 0 & 0 & 0 & 0 & 0 & d_{36} \end{pmatrix}$$

With d_{36} = 370 pm/V. The nanowire was grown in the (111) direction so it can lie on one of its three different crystal planes, for example the (-110) facets (see Figure S7). We define the x-axis as the (111) growth direction, the y-direction in the first case as the (11-2) crystal plane, in the

second case as (01-1) and in the third case as (-12-1). The z-direction corresponds then to the (-110), (-211) or (-101) crystal plane.

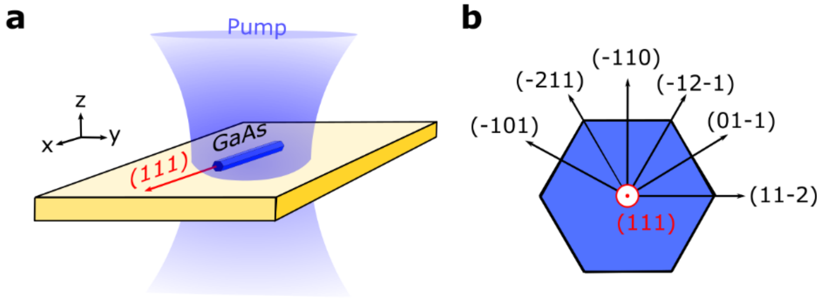

Figure S7: (a) Schematic of the SPDC process, with the lying NW and the (111) growth axis, which we will label the x-direction. (b) The Nanowire can lie on three different crystal planes.

If $\{e_i\}_{i=1,2,3}$ and $\{f_a\}_{a=1,2,3}$ are two orthonormal frames and $\boldsymbol{\mathcal{R}}$ is the linear map such that $\boldsymbol{\mathcal{R}}e_1 = f_1$, $\boldsymbol{\mathcal{R}}e_2 = f_2$ and $\boldsymbol{\mathcal{R}}e_3 = f_3$ (i.e., $\boldsymbol{\mathcal{R}}_{ia} = f_a \cdot e_i$) then the components of a vector with respect to the $\{f_a\}_{a=1,2,3}$ are related to the components of the same vector with respect to $\{e_i\}_{i=1,2,3}$ through $v_a = \boldsymbol{\mathcal{R}}_{ia}v_i$. For third-order tensors (as $\chi^{(2)}$) a similar rule applies and the components with respect to the $\{f_a\}_{a=1,2,3}$ frame are related to the components with respect to $\{e_i\}_{i=1,2,3}$ frame through:

$$\chi_{abc}^{(2)} = \mathcal{R}_{ia}\mathcal{R}_{jb}\mathcal{R}_{kc}\chi_{ijk}^{(2)} \qquad (1)$$

The classical relation $P = \varepsilon_0\chi^{(2)}[E, E]$ (or using the components:

$P_i = \varepsilon_0\chi_{ijk}^{(2)}E_jE_k$ can be formally written with the $\chi^{(2)}$ tensor in d-notation as

$$\begin{pmatrix} P_x \\ P_y \\ P_z \end{pmatrix} = \begin{pmatrix} d_{11} & d_{12} & d_{13} & d_{14} & d_{15} & d_{16} \\ d_{16} & d_{22} & d_{23} & d_{24} & d_{14} & d_{12} \\ d_{15} & d_{24} & d_{33} & d_{23} & d_{13} & d_{14} \end{pmatrix} \begin{pmatrix} E_x^2 \\ E_y^2 \\ E_z^2 \\ 2E_yE_z \\ 2E_zE_x \\ 2E_xE_y \end{pmatrix},$$

where

$$
\begin{aligned}
&d_{11} = \chi^{(2)}_{xxx}, \quad d_{22} = \chi^{(2)}_{yyy}, \quad d_{33} = \chi^{(2)}_{zzz}, \\
&d_{12} = \chi^{(2)}_{xyy} = \chi^{(2)}_{yxy} = \chi^{(2)}_{yyx}, \quad d_{13} = \chi^{(2)}_{xzz} = \chi^{(2)}_{zxz} = \chi^{(2)}_{zzx}, \\
&d_{23} = \chi^{(2)}_{yzz} = \chi^{(2)}_{zyz} = \chi^{(2)}_{zzy}, \quad d_{16} = \chi^{(2)}_{xxy} = \chi^{(2)}_{xyx} = \chi^{(2)}_{yxx}, \\
&d_{24} = \chi^{(2)}_{zyy} = \chi^{(2)}_{yzy} = \chi^{(2)}_{yyz}, \quad d_{15} = \chi^{(2)}_{xxz} = \chi^{(2)}_{xzx} = \chi^{(2)}_{zxx}, \\
&d_{14} = \chi^{(2)}_{xyz} = \chi^{(2)}_{xzy} = \chi^{(2)}_{yzx} = \chi^{(2)}_{yxz} = \chi^{(2)}_{zxy} = \chi^{(2)}_{zyx.}.
\end{aligned}
\tag{2}
$$

The cubic symmetry of the crystal with respect to the crystal axes (i.e., [100], [010] and [001]) impose

$$
D = d_{14}\begin{pmatrix} 0 & 0 & 0 & 1 & 0 & 0 \\ 0 & 0 & 0 & 0 & 1 & 0 \\ 0 & 0 & 0 & 0 & 0 & 1 \end{pmatrix}
$$

and, in order to obtain the components of $\chi^{(2)}$ (in d-notation), we first compute the components of the $\chi^{(2)}$ tensor using relation (1) and then use relation (2). A Mathematica Script is provided attached. As an illustration, in order to compute the components of the $\chi^{(2)}$ (in d-notation) with respect to the orthonormal frame

$$
f_1 = \frac{1}{\sqrt{3}}\begin{pmatrix} 1 \\ 1 \\ 1 \end{pmatrix}, f_2 = \frac{1}{\sqrt{6}}\begin{pmatrix} 1 \\ 1 \\ -2 \end{pmatrix}, f_3 = \frac{1}{\sqrt{2}}\begin{pmatrix} -1 \\ 1 \\ 0 \end{pmatrix}, \tag{3}
$$

We use in (1)

$$
R^{(1)} = \begin{pmatrix} \frac{1}{\sqrt{3}} & \frac{1}{\sqrt{6}} & -\frac{1}{\sqrt{2}} \\ \frac{1}{\sqrt{3}} & \frac{1}{\sqrt{6}} & \frac{1}{\sqrt{2}} \\ \frac{1}{\sqrt{3}} & -\frac{\sqrt{2}}{\sqrt{3}} & 0 \end{pmatrix}, \tag{4}
$$

and obtain for $\chi^{(2)}$ (in d-notation

$$
\chi^{(2,1)} = d_{14}\begin{pmatrix} \frac{2}{\sqrt{3}} & -\frac{1}{\sqrt{3}} & -\frac{1}{\sqrt{3}} & 0 & 0 & 0 \\ 0 & -\sqrt{\frac{2}{3}} & \sqrt{\frac{2}{3}} & 0 & 0 & -\frac{1}{\sqrt{3}} \\ 0 & 0 & 0 & \sqrt{\frac{2}{3}} & -\frac{1}{\sqrt{3}} & 0 \end{pmatrix}. \tag{5}
$$

The two other cases are

$$R^{(2)} = \begin{pmatrix} \frac{1}{\sqrt{3}} & 0 & -\sqrt{\frac{2}{3}} \\ \frac{1}{\sqrt{3}} & \frac{1}{\sqrt{2}} & \frac{1}{\sqrt{6}} \\ \frac{1}{\sqrt{3}} & -\frac{1}{\sqrt{2}} & \frac{1}{\sqrt{6}} \end{pmatrix}, \quad (6)$$

which gives

$$\chi^{(2,2)} = d_{14} \begin{pmatrix} \frac{2}{\sqrt{3}} & -\frac{1}{\sqrt{3}} & -\frac{1}{\sqrt{3}} & 0 & 0 & 0 \\ 0 & 0 & 0 & \sqrt{\frac{2}{3}} & 0 & -\frac{1}{\sqrt{3}} \\ 0 & \sqrt{\frac{2}{3}} & -\sqrt{\frac{2}{3}} & 0 & -\frac{1}{\sqrt{3}} & 0 \end{pmatrix}. \quad (7)$$

And

$$R^{(3)} = \begin{pmatrix} \frac{1}{\sqrt{3}} & -\frac{1}{\sqrt{6}} & -\frac{1}{\sqrt{2}} \\ \frac{1}{\sqrt{3}} & \sqrt{\frac{2}{3}} & 0 \\ \frac{1}{\sqrt{3}} & -\frac{1}{\sqrt{6}} & \frac{1}{\sqrt{2}} \end{pmatrix}, \quad (8)$$

for which

$$\chi^{(2,2)} = d_{14} \begin{pmatrix} \frac{2}{\sqrt{3}} & -\frac{1}{\sqrt{3}} & -\frac{1}{\sqrt{3}} & 0 & 0 & 0 \\ 0 & \sqrt{\frac{2}{3}} & -\sqrt{\frac{2}{3}} & 0 & 0 & -\frac{1}{\sqrt{3}} \\ 0 & 0 & 0 & -\sqrt{\frac{2}{3}} & -\frac{1}{\sqrt{3}} & 0 \end{pmatrix}. \quad (9)$$

We recorded the SHG for different polarizations of the pump laser for the 14 NWs, as shown in Figure S8. We always observed the strongest SHG signal for laser light polarized in the x-direction (along the NW), which is in accordance with the calculations, as the $\chi^{(2,i)}_{x,x,x}$ is non-zero for every rotation frame i=1,2,3. Since the laser was polarized in x-direction and was not tightly focused, we assumed to have a dominant x-component of the electric field at the NW and only a small y- and

z-component. We conclude that especially the $\chi^{(2)}_{x,x,x}$ is a key factor to explain the nonlinear signal generation.

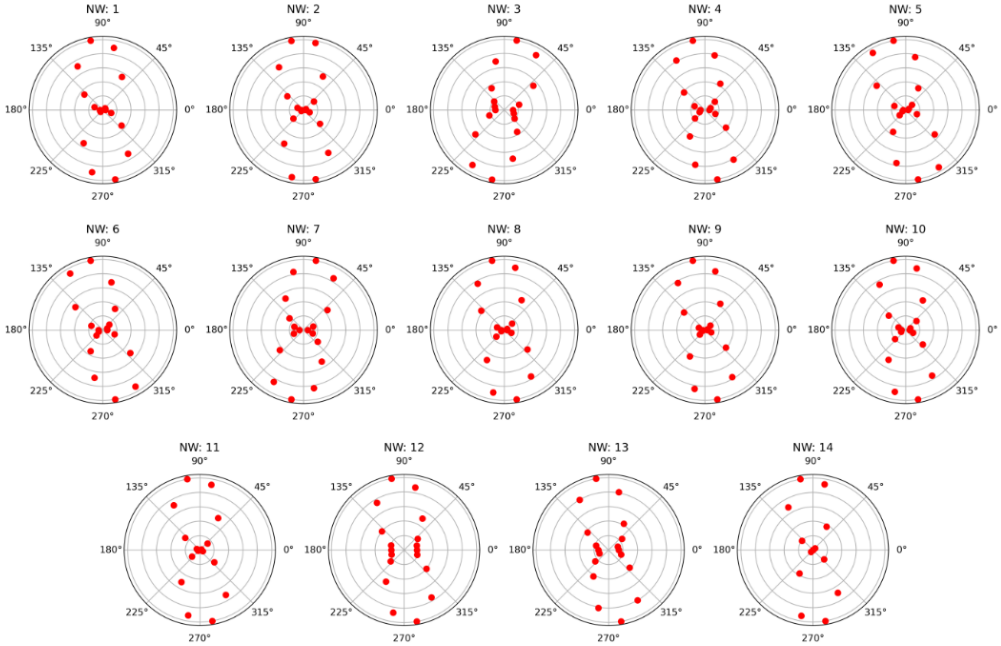

Figure S8: SHG intensity for each NW with respect to the linear polarizations orientation of the pump. All Nanowire show dipolar-shaped dependence, with maximum SHG intensity with polarization along the NW.

6. Summary SPDC efficiency calculation

We summarize in Table S1 all the results to compute the SPDC efficiency of each NWs. This is calculated by normalizing the measured SPDC rate with the transmission, the pump fluence and the nanowire volume. We take into account the fixed 0.14% transmission in the setup for a photon-pair, as explained in section 3 of the supplementary material. The pump fluence is also measured as 0.5 mW/μm^2. By comparing the NWs sizes before (5.2 ± 0.4 μm) and after (4.1 ± 1.0 μm) mechanical transfer, we deduce that the bottom of the NWs breaks. The diameter is still in the

expected range of 430 ± 30 nm. The sizes and SPDC efficiencies of each NWs is also shown in Figure S9.

Table S1: SPDC efficiency calculations for all 14 different single NWs.

NW no	Length [μm]	Diameter [nm]	Measured Coincidences [mHz]	Corrected Biphoton rate [Hz]	SPDC Efficiency [GHz/Wm]
1	4.9	440	27	19.3	58.9
2	4.5	370	13	9.6	45.2
3	5.0	400	9	6.7	24.3
4	4.6	400	11	7.9	31.3
5	3.3	370	12	8.6	55.3
6	5.3	430	11	7.5	22.3
7	5.3	415	7	5.1	16.3
8	3.8	390	13	9.1	45.9
9	4.4	350	7	4.8	25.8
10	5.4	440	8	5.4	15.0
11	3.5	300	6	4.1	37.3
12	2.5	290	4	2.5	34.9
13	2.4	325	3	1.8	20.9
14	2.7	320	3	1.9	19.9
Average	4.1±1.0	375±50			32±14

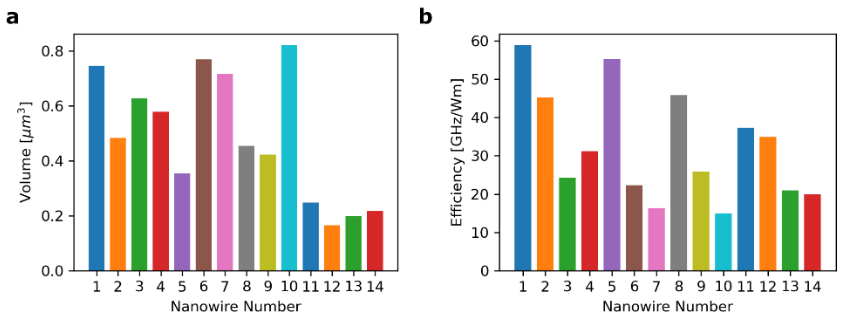

Figure S9: (a) Volumes and (b) SPDC efficiencies for the 14 different Nanowires.

In order to understand the SPDC intensity variations for the different NWs, we performed an in-depth analysis of the correlation between the biphoton rate and the SHG intensity, as shown in Figure 4a, and the NWs' volume, length and diameter, see Figure S10. We calculated the Pearson coefficient $c = \frac{COV(x,y)}{\sigma_x \sigma_y}$, with COV the covariance and σ the standard deviation. We observed that

the biphoton rate was clearly correlated with the SHG intensity, but that it showed no strong dependency on the volume, the length nor the diameter of the NW. Moreover, we calculated the correlation of the biphoton rate with the linear scattering intensity at the pump wavelength and at the photon pair wavelength. The linear scattering intensity at 775 nm was not correlated at all with the nonlinear emission, while the correlation with the linear scattering intensity at 1550 nm seemed very weak.

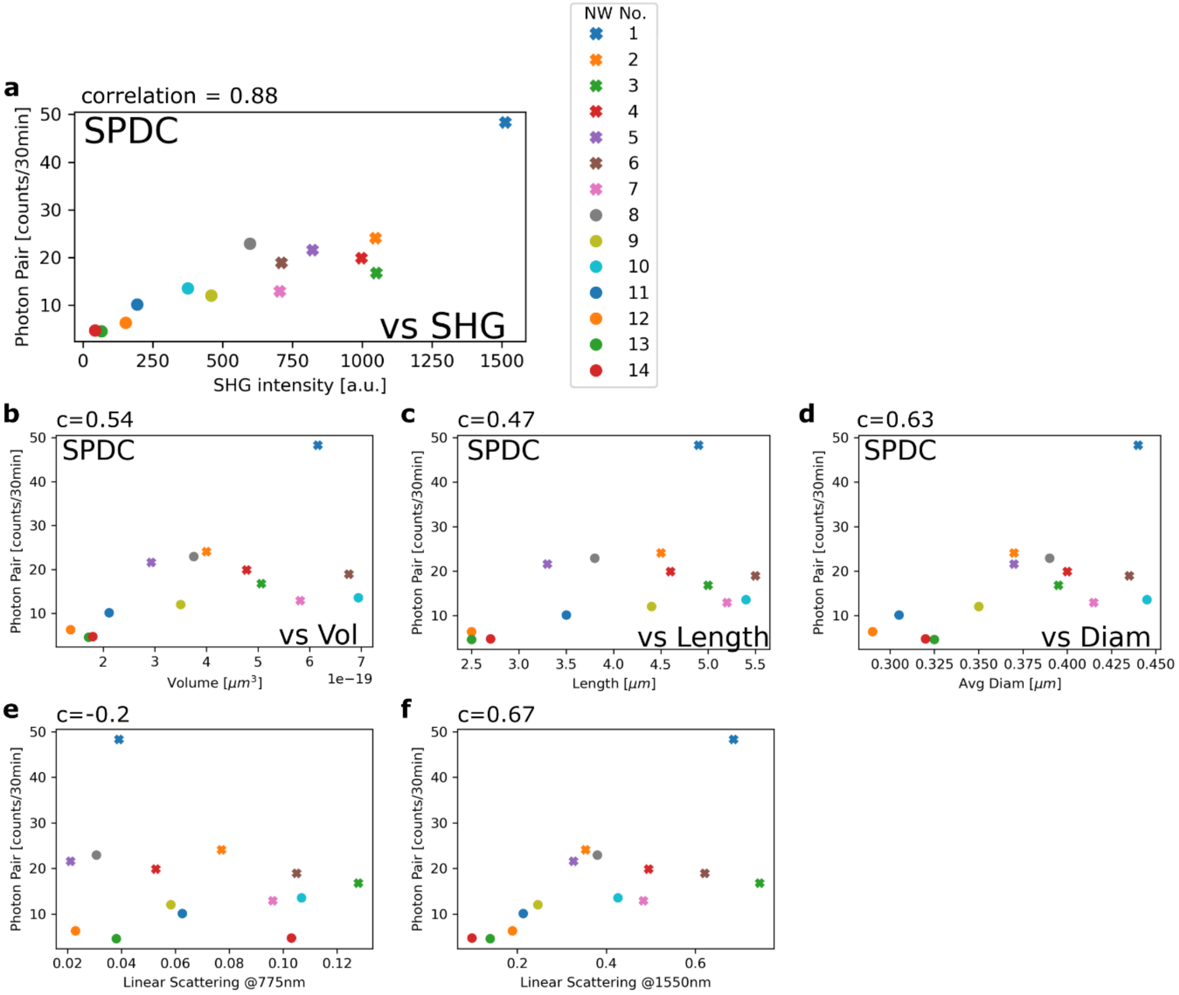

Figure S10. Correlation measurements for the 14 NWs between the biphoton rate and (a) the SHG intensity they emit (same as Figure 4a), (b) their volume, (c) their length and their diameter. Additionally, we measured the linear scattering intensity (e) at the excitation (775 nm) and (f) at

photon pair wavelengths (1550 nm) and correlated these values to the biphoton rates, similarly as in (a)-(d). The correlation values between -1 (perfectly anti-correlated) and 1 (perfectly correlated) are given above each graph. The SPDC intensity was strongly correlated to the SHG, while the three size parameters (volume, length and diameter) seemed to have a minor impact on the nonlinear emission. Concerning the linear scattering, the SPDC intensity was slightly correlated to the linear scattering intensity at 1550 nm and not at all to the one at 775 nm, see (e) and (f).

The overall crystal structure uniformity is shown in Figure S3-S4, but nanoscopic crystal structure defects or doping concentration variations may be present and could still have some impact on the biphoton emission efficiency.

One could measure the photon purity in a heralded single photon HBT setup by analyzing one of the photons, similarly as in single photon experiments. Due to the energy conservation, if the SPDC process is pumped at 775 nm, one of the photon has a wavelength above 1550 nm and one below. The lower (higher) energy photon can be isolated in one of the arms by adding a low-pass (high-pass) filter, while the higher (lower) energy photon is blocked in the other arm. Statistically, 50% of photon pairs generated from the NWs would lead to a single photon, and measuring the dip in the g(2) function can give information on the photon purity. Further characterization of the photon pairs from Type 0 SPDC is thus possible, even though they cannot be deterministically split using simply a polarized beam splitter as in common Type II SPDC.

REFERENCES

(1) Fontcuberta I Morral, A.; Spirkoska, D.; Arbiol, J.; Heigoldt, M.; Morante, J. R.; Abstreiter, G. Prismatic Quantum Heterostructures Synthesized on Molecular-Beam Epitaxy GaAs Nanowires. *Small* **2008**, *4* (7), 899–903.

(2) Wagner, R. S.; Ellis, W. C. Vapor-Liquid-Solid Mechanism of Single Crystal Growth. *Appl. Phys. Lett.* **1964**, *4* (5), 89–90.

(3) Küpers, H.; Bastiman, F.; Luna, E.; Somaschini, C.; Geelhaar, L. Ga Predeposition for the Ga-Assisted Growth of GaAs Nanowire Ensembles with Low Number Density and Homogeneous Length. *J. Cryst. Growth* **2017**, *459*, 43–49.

(4) Fouquat, L.; Vettori, M.; Botella, C.; Benamrouche, A.; Penuelas, J.; Grenet, G. X-Ray Photoelectron Spectroscopy Study of Ga Nanodroplet on Silica-Terminated Silicon Surface for Nanowire Growth. *J. Cryst. Growth* **2019**, *514*, 83–88.

(5) Rudolph, D.; Hertenberger, S.; Bolte, S.; Paosangthong, W.; Spirkoska, D.; Döblinger, M.; Bichler, M.; Finley, J. J.; Abstreiter, G.; Koblmüller, G. Direct Observation of a Noncatalytic Growth Regime for GaAs Nanowires. *Nano Lett.* **2011**, *11* (9), 3848–3854.

(6) Saerens, G.; Tang, I.; Petrov, M. I.; Frizyuk, K.; Renaut, C.; Timpu, F.; Reig Escalé, M.; Shtrom, I.; Bouravleuv, A.; Cirlin, G.; et al. Engineering of the Second-Harmonic Emission Directionality with III–V Semiconductor Rod Nanoantennas. *Laser Photonics Rev.* **2020**, *14* (9), 1–10.

(7) Timofeeva, M.; Lang, L.; Timpu, F.; Renaut, C.; Bouravleuv, A.; Shtrom, I.; Cirlin, G.; Grange, R. Anapoles in Free-Standing III-V Nanodisks Enhancing Second-Harmonic Generation. *Nano Lett.* **2018**, *18* (6), 3695–3702.

(8) Timpu, F.; Sendra, J.; Renaut, C.; Lang, L.; Timofeeva, M.; Buscaglia, M. T.; Buscaglia, V.; Grange, R. Lithium Niobate Nanocubes as Linear and Nonlinear Ultraviolet Mie Resonators. *ACS Photonics* **2019**, *6* (2), 545–552.

(9) Boyd, R. W. *Nonlinear Optics*; Elsevier Science Publishing Co Inc, 2008, pp 1-67.
